# Performance of three molecular methods for detection of *Toxoplasma gondii* in pork

**DOI:** 10.1016/j.fawpar.2019.e00038

**Published:** 2019-02-08

**Authors:** Nadja S. Bier, Gereon Schares, Annette Johne, Annett Martin, Karsten Nöckler, Anne Mayer-Scholl

**Affiliations:** a German Federal Institute for Risk Assessment (BfR), Department for Biological Safety, Berlin, Germany; b Friedrich-Loeffler-Institut, Federal Research Institute for Animal Health, Greifswald, Insel Riems, Germany; c German Federal Institute for Risk Assessment (BfR), Department for Exposure, Berlin, Germany

**Keywords:** Pork, qPCR, 529 RE, Food safety

## Abstract

Comparison of epidemiological data on the occurrence of *Toxoplasma (T.) gondii* tissue cysts in meat is hampered by the lack of standardization and a great variety of methods for molecular detection. Therefore, this study aimed to compare and validate three different polymerase chain reaction (PCR) methods for detection of *T. gondii* DNA in pork. Analytical performance characteristics of two real time PCRs (qPCRs; Tg-qPCR1, Tg-qPCR2) and one conventional endpoint PCR (cPCR), all targeting the 529 repeated element, were assessed using genomic DNA of three clonal *T. gondii* types prevailing in Europe and North America. qPCR efficiencies for all three clonal types ranged between 93.8 and 94.4% (Tg-qPCR1) and 94.3–95.6% (Tg-qPCR2). Tg-qPCR1 and Tg-qPCR2 showed an overall PCR performance score of 85% and displayed a similar 95% detection limit of 1.067 and 1.561 genome equivalents per PCR reaction (GE/PCR), respectively. However, *T. gondii* DNA could be detected at concentrations as low as 0.1 GE/PCR. Reliable quantification is possible over 4 log ranges from 10^5^ to 100 GE/PCR with mean repeatability relative standard deviations of ≤11% and reproducibility relative standard deviations of ≤12.7%. Presumably, both qPCRs are similarly suitable for sensitive and specific detection of *T. gondii* DNA in pork. In contrast, the cPCR using primer pair TOX5/Tox-8 proved to be highly sensitive with a detection limit of 1.41 GE/PCR, but not suitable for detection of *T. gondii* DNA in pork as unspecific amplification of porcine DNA was observed resulting in bands with similar size to the desired *T. gondii*-specific PCR product.

## Highlights

• Comparison of two real time PCRs and one conventional PCR targeting the 529 RE.
• Both real time PCRs show comparable performance and can be considered equivalent.
• qPCRs show 95% detection limits of 1.067 and 1.561 genome equivalents per PCR.
• Reliable quantification is possible over 4 log ranges from 10^5^ to 100 GE/PCR.
• cPCR using primer pair TOX5/Tox-8 showed non-specific amplification of porcine DNA.

## 1 Introduction

Toxoplasmosis is one of the most common parasitic zoonosis worldwide with approximately 30% of the human population chronically infected (Robert-Gangneux and Darde, 2012). Based on a high disease burden (expressed in Disability Adjusted Life Years), *Toxoplasma* (*T.*) *gondii* globally ranks among the most important foodborne pathogens (Scallan et al., 2015; WHO, 2015). Toxoplasmosis can be acquired horizontally by smear infection or oral ingestion of infectious parasite stages such as consumption of raw or undercooked meat containing highly infectious *T. gondii* tissue cysts. Water or fresh fruits and vegetables contaminated with *T. gondii* oocysts represent an additional infection source (Tenter et al., 2000). However, consumption of raw meat is considered as one of the most important risk factors for *T. gondii* infection in Europe (Kapperud et al., 1996; Cook et al., 2000).

For the assessment of meaningful and representative data on the occurrence of *T. gondii* tissue cysts in meat, reliable and sensitive methods for molecular detection are essential. However, comparison of epidemiological data is hampered by the great variety of methods for molecular detection of *T. gondii* described in the literature and lacking standardization. Published molecular techniques include conventional endpoint polymerase chain reaction (PCR), nested PCR, real time PCR (qPCR, either using SYBR green or TaqMan™ probes), and amplified loop-mediated isothermal amplification (LAMP) protocols which are reviewed elsewhere (Liu et al., 2015; Dzib Paredes et al., 2016). Moreover, several target genes have been used to detect *T. gondii* DNA in different sample types. Some target genes are present in multicopy, such as the B1 gene, the 529 repeated element (RE), the internal transcribed spacer element ITS-1, and 18S rDNA, while other targets represent single-copy genes such as the genes coding for the GRA1, GRA6, SAG1, and SAG2 proteins. In addition, there is also a diversity of published primers and protocols for the same molecular technique using an identical target gene.

This study aimed to compare the analytical and diagnostic performance of one conventional endpoint PCR (cPCR) and two qPCR assays for detection of *T. gondii* DNA in pork meat samples. The *T. gondii* (Tg)-qPCR1 (Opsteegh et al., 2010) and Tg-qPCR2 (Talabani et al., 2009) as well as a cPCR (Schares et al., 2008) using the primers TOX5 (Homan et al., 2000) and Tox-8 (Reischl et al., 2003) were chosen, as they are used in different reference laboratories across Europe and described in various studies (Schares et al., 2008; Herrmann et al., 2010; Herrmann, 2012; Gomez-Samblas et al., 2015; Opsteegh et al., 2016; Krücken et al., 2017; Schares et al., 2017; Burrells et al., 2018; Friedrich-Loeffler-Institut, 2018; Schares et al., 2018). Moreover, all three assays target the 529 RE that shows up to 200 to 300 copies per genome and thus generally allows detection of *T. gondii* DNA with a higher sensitivity compared to other targets genes such as the 35-copy B1 gene (Sterkers et al., 2010). As the number of repeats can vary among different *T. gondii* strains (Costa and Bretagne, 2012), performance characteristics were assessed using representative strains of the three clonal types prevailing in Europe and North America.

## 2 Materials & methods

### 2.1 Reference material

Genomic DNA of strains representative of the three clonal *T. gondii* types (strains RH (type 1), ME-49 (type 2), and NED (type 3)) served as reference material and was extracted from ~10^9^ tachyzoites cultivated in MARC-145 cell monolayers. Tachyzoite pellets were incubated in 1 ml lysis buffer (100 mM sodium chloride (NaCl), 25 mM ethylene diamine tetraacetic acid, 0.5% sodium dodecyl sulphate, 10 mM Tris pH 8, 0.1 mg/ml proteinase K) for 12 h at 55 °C. DNA was extracted several times with 1 vol phenol-chloroform-isoamylalcohol (in a 25:24:1 ratio) until the supernatant was without visible contaminants and collected in a fresh 1.5 ml reaction tube. 4 M NaCl was added to yield a final 0.2 M NaCl concentration. DNA was precipitated with 2 vol absolute ethanol, washed with 1 ml 70% (v/v) ethanol, dried, resuspended in 100 μl of molecular grade water and stored at 4 °C. DNA extraction of tachyzoites of *Neospora caninum* and *Besnoitia besnoiti* was performed analogously. For *Hammondia hammondi* and *Hammondia heydorni*, DNA extraction was performed from oocysts using the NucleoSpin® Soil Kit (MACHEREY-NAGEL GmbH, Germany) according to the manufacturer's instructions. The quality and concentration of DNA extracts were determined using the NanoDrop 1000 Spectrophotometer (Peqlab, Erlangen, Germany). Based on the published genome size of 65.67 Mb of *T. gondii* strain ME-49 (GenBank Assembly ID GCA_000006565.2.) and a weight of 650 Da for 1 bp, one haploid genome of *T. gondii* was considered equivalent to 70.88 fg.

### 2.2 529 RE quantitative polymerase chain reaction (qPCR)

Tg-qPCR1 and Tg-qPCR2 were carried out as described previously with some modifications (Talabani et al., 2009; Opsteegh et al., 2010). Reactions were performed on a 7500 real-time PCR system (Applied Biosystems, ABI) in a total volume of 25 μl using 10 μl template DNA and 12.5 μl of 2× TaqMan™ universal PCR mastermix (ABI). Oligonucleotides were synthesized by Metabion International AG (Martinsried, Germany) or TIB MOLBIOL Syntheselabor GmbH (Berlin, Germany) and are used at final concentrations listed in Table 1.

**Table 1:** Oligonucleotide primers and TaqMan™ probes.

Assay	Primer name	Gene target	Sequence (5′ - 3′)^a^	Cycling conditions	Final concentration in PCR (μM)	Reference
cPCR	Tox-8	*T. gondii 529* RE	CCCAGCTGCGTCTGTCGGGAT	94 °C - 1 min 35×: 94 °C - 1 min 60 °C - 1 min 72 °C - 1 min 72 °C - 10 min	0.5	(Reischl et al., 2003, Schares et al., 2008, Herrmann, 2012)
	TOX5	*T. gondii 529* RE	CGCTGCAGACACAGTGCATCTGGATT		0.5	(Homan et al., 2000, Schares et al., 2008, Herrmann, 2012)

Tg-qPCR1	Tox-9F	*T. gondii 529* RE	AGGAGAGATATCAGGACTGTAG	50 °C - 2 min 95 °C - 10 min 45×: 95 °C -15 s 58 °C - 20 s 72 °C - 30 s	0.7	(Reischl et al., 2003, Opsteegh et al., 2010)
	Tox-11R	*T. gondii 529* RE	GCGTCGTCTCGTCTAGATCG		0.7	(Reischl et al., 2003, Opsteegh et al., 2010)
	Tox-TP1	*T. gondii 529* RE	FAM™-CCGGCTTGGCTGCTTTTCCT-BHQ-1		0.1	(Opsteegh et al., 2010)
	CIAC-probe	*Yersinia pestis caf1*	JOE™-AGCGTACCAACAAGTAATTCTGTATCGATG-BHQ-1		0.2	(Opsteegh et al., 2010)

Tg-qPCR2	*T. gondii* forward	*T. gondii 529* RE	TGG TTG GGA AGC GAC GAG AG	50 °C - 2 min 95 °C - 10 min 55×: 95 °C - 15 s 60 °C - 15 s 72 °C - 30 s	0.8	(Talabani et al., 2009)
	*T. gondii* reverse	*T. gondii 529* RE	CAT CAC CAC GAG GAA AGC GTC		0.8	(Talabani et al., 2009)
	*T. gondii* LNA probe	*T. gondii 529* RE	FAM™-AG [+A]GA [+C]AC [+C]GG [+A]ATGCG [+A]T-BHQ-1		0.2	(Talabani et al., 2009)
	pUC 18-F	pUC18/19	TGT CGT GCC AGC TGC ATT A		0.075	(Mäde et al., 2008, Anonymous, 2013, Frentzel et al., 2018)
	pUC 18-R	pUC18/19	GAG CGA GGA AGC GGA AGA G		0.075	(Mäde et al., 2008, Anonymous, 2013, Frentzel et al., 2018)
	Tm-pUC18	pUC18/19	JOE™-AAT CGG CCA ACG CGC GG-BHQ-1		0.1	(Mäde et al., 2008, Anonymous, 2013, Frentzel et al., 2018)

a TaqMan™ probes are labelled with reporter dyes (5′-end) and quenchers (3′-end): FAM™, 6-carboxyfluorescein; JOE™, 5-carboxy-4′,5′-dichloro-2′,7′-dimethoxyfluorescein; BHQ, Black Hole Quencher; LNA probe, locked nucleic acid-substituted TaqMan™ probe.

In order to identify false-negative results due to PCR inhibition, internal amplification controls (IAC) were included in both qPCRs. For Tg-qPCR1, a PCR product (189 bp) based on the *Yersinia pestis caf1* gene was generated as previously described (Opsteegh et al., 2010) and used as a competitive IAC. However, the CIAC was used at a lower final concentration of 0.008 fg (40 copies) per qPCR reaction to ensure stable CIAC amplification with Cq values of 33–35 and to reduce inhibition of *T. gondii* DNA amplification to a minimum. In Tg-qPCR2, pUC18 DNA (Thermo Fisher Scientific) was included as a non-competitive IAC at a final concentration of 1.45 fg (500 copies) per qPCR reaction to enable stable amplification with Cq values of 31–33. Cycling conditions for both qPCR assays are listed in Table 1. In each run, a non-template control was included and genomic DNA of *T. gondii* strain RH corresponding to 100 GE/reaction served as positive control.

### 2.3 Analysis of qPCR runs

The 7500 Fast Systems Software (ABI) was used to calculate the quantification cycle (Cq). After adjusting the threshold line for each target and each run independently in the middle of the exponential phase, the mean threshold ΔRn value over all runs was calculated and evaluated as the optimal threshold value for the corresponding target gene. For standardization during the validation process, the threshold lines were set manually to the established ΔRn values of 0.7 (529 RE, Tg-qPCR1), 0.08 (529 RE, Tg-qPCR2), 0.03 (CIAC, Tg-qPCR1), and 0.03 (pUC18, Tg-qPCR2), respectively.

All samples which showed exponential amplification with a Cq value <40 were scored positive for *T. gondii* DNA. Samples without exponential amplification or a Cq value ≥40 were scored negative, if amplification of the internal amplification control (IAC) could be observed.

### 2.4 Analytical specificity and sensitivity of qPCR assays

The specificity of the assays was determined using 1 ng of DNA of related parasitic species belonging to the subfamily of *Toxoplasmatinae* including *Neospora caninum*, *Besnoitia besnoiti*, *Hammondia hammondi*, and *Hammondia heydorni*.

The analytical sensitivity of both qPCR assays was determined by serially diluting genomic *T. gondii* DNA of strains representative of the three clonal types to obtain 10^5^, 10^4^, 10^3^, 10^2^, 10, 1, 0.75, 0.5, 0.25, and 0.1 *T. gondii* genome equivalents (GE) per PCR reaction. The dilution was prepared in water containing 20 ng/μl porcine DNA resulting in a background of 200 ng matrix DNA per PCR reaction when using 10 μl samples as template DNA (BVL, 2016). Dilution series were freshly prepared before each run and examined with both qPCRs on the same day. Serial dilutions were analyzed in triplicates in four qPCR runs resulting in 12 replicate measurements for each dilution point.

The 95% detection limit was estimated by probit analysis (IBM SPSS Statistics 21). A PCR performance score was determined by dividing the number of positive replicates by the total number of replicates (Robert-Gangneux et al., 2017).

### 2.5 Precision, PCR efficiency & limit of quantification (LOQ) of qPCR assays

Standard curves were generated based on tenfold dilution series from 10^5^ GE to 10 GE by plotting the mean Cq-value in one run against the logarithm of the template concentration as the GE number per PCR reaction. The slope (s) of the corresponding linear regression line was used to calculate the PCR efficiency E with the formula E = 10^–1/s^ − 1 (Bustin et al., 2009). PCR efficiencies were calculated for each run and clonal type DNA (triplicates).

To evaluate the precision, measured Cq values of each replicate were converted into the actual template concentration using the corresponding standard curves. Based on these back calculated concentrations the coefficient of variation (CV(%) = 100× SD/mean) was calculated for each dilution point (Bustin et al., 2009; Kralik and Ricchi, 2017). To evaluate intra-assay variance, the relative repeatability standard deviation (Rsd_r_) was determined as the CV of each dilution point in each run.

To evaluate the inter-assay variance, the relative reproducibility standard deviation (Rsd_R_) was determined as the CV of each dilution point over different runs (Bustin et al., 2009; Broeders et al., 2014). The limit of quantification (LoQ) was defined as the lowest concentration at which replicates over four runs show a mean Rsd_r_ ± SD ≤25% on back calculated GE numbers (Kralik and Ricchi, 2017).

### 2.6 Performance of the Tg-qPCR1 and Tg-qPCR2 assays on pork meat samples

To assess the performance of both qPCR assays using field samples, 38 pork meat products from conventionally raised pigs (35 meat cutlets, 2 minced meat, 1 sausage) were purchased at retail markets and subjected to DNA extraction and examination by both qPCR assays. To generate *T. gondii*-positive samples, 25 mg portions of 12 negatively tested samples were spiked with a low (100 GE/25 mg sample, n = 4), medium (1000 GE/25 mg sample, n = 4), and high (10,000 GE/25 mg sample, n = 4) amount of *T. gondii* DNA (strain ME-49) before DNA extraction.

DNA was extracted from 25 mg meat using the QIAamp DNA Mini Kit (Qiagen) according to the manufacturer's instructions. DNA was eluted with 100 μl of molecular grade water and stored at 4 °C. In each experiment a negative extraction control using 25 μl molecular grade water was included to exclude possible cross-contaminations.

### 2.7 529 RE conventional endpoint PCR analysis

The cPCR was performed similar to a previously described cPCR (Schares et al., 2008; Herrmann, 2012; Friedrich-Loeffler-Institut, 2018). Amplifications were carried out on a 2720 Thermal Cycler (ABI) in a total volume of 25 μl with 1× DreamTaq buffer (2 mM MgCl_2_), 0.25 mM of each deoxynucleoside triphosphate, 0.5 μM of each of the primers TOX5 and Tox-8 (Table 1), 20 μg/ml bovine serum albumin (New England Biolabs), 1 U DreamTaq DNA Polymerase (Thermo Fisher Scientific) and 10 μl template DNA. 10 μl PCR product were separated by electrophoresis on 1.2% agarose and visualized using GelRed® in a final concentration of 0.008% (v/v) (Biotium).

## 3 Results

### 3.1 Analytical validation of qPCR assays

#### 3.1.1 Specificity

Neither the Tg-qPCR1 nor Tg-qPCR2 showed cross-reaction with DNA of related non-target species, confirming the analytical specificity of both assays.

#### 3.1.2 Sensitivity & limit of detection (LoD)

In both qPCR assays, *T. gondii* DNA could be detected at concentrations as low as 0.1 GE/PCR with mean Cq values between 34.1 and 38.1 (Tg-qPCR1) and 35.1–38.2 (Tg-qPCR2) for all three clonal type strains (Table 2). Considering the results of all three types, both qPCRs showed an identical PCR performance score of 85% (Table 3) and a similar 95% detection limit of 1.067 GE (95% CI: 0,773-1789) and 1.561 GE (95% CI: 0,998-3506) for Tg-qPCR1 and Tg-qPCR2, respectively. The probit regression model fitted the data adequately (Tg-qPCR1: Pearson χ^2^ = 3637, p = 0.89; Tg-qPCR2: Pearson χ^2^ = 6.454, p = 0.597) (Table 3). However, Tg-qPCR2 showed a slightly higher sensitivity, as it was able to detect a higher percentage of replicates (39%, 14/36) at low concentrations of 0.1 GE/PCR compared to Tg-qPCR1, which was positive in only 19% (7/36) of the replicates (Table 2, Table 3). Interestingly, DNA of *T. gondii* NED (type 3) could be detected with higher sensitivity compared to the other two clonal type strains (Table 2, Table 3). As all strains showed nearly identical Cq values at higher DNA concentrations, this phenomenon cannot be explained by differences in the DNA concentration or by different numbers of the 529 RE repeats.

**Table 2:**
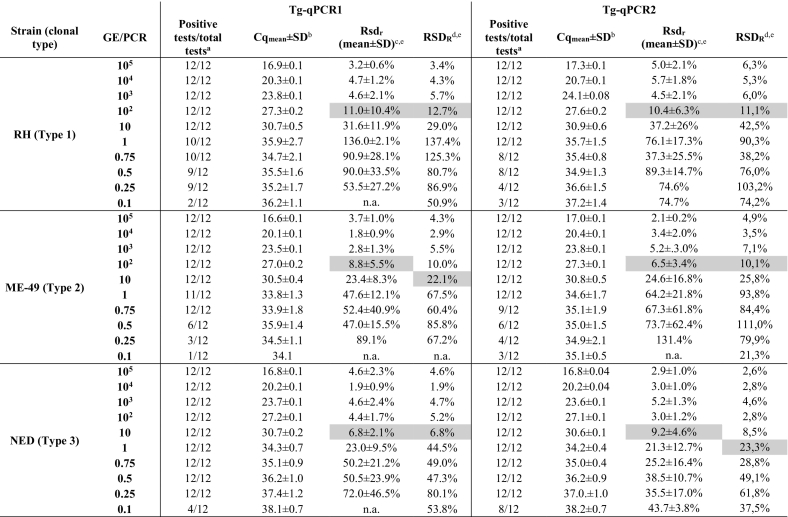
Analytical sensitivity and precision of Tg-qPCR1 and Tg-qPCR2 for each of the three clonal types of *Toxoplasma gondii* prevailing in Europe and North-America.

GE/PCR, number of genome equivalents per PCR reaction; Cq, quantification cycle; SD, standard deviation; n.a., not applicable. ^a^ 12 replicates were obtained by testing triplicates in four runs. ^b^ Mean Cq values of 12 replicates and standard deviations are shown. ^c^ Rsd_r_, relative repeatability standard deviation calculated for each run and averaged over four runs. ^d^ Rsd_R_, relative reproducibility standard deviations calculated over four runs. ^e^ The lowest concentrations with acceptable Rsd_r_ and Rsd_R_ values ≤25% are highlighted in grey.

**Table 3:** Performance characteristics of Tg-qPCR1 and Tg-qPCR2 for each of the three clonal types of *Toxoplasma gondii* prevailing in Europe and North-America.

Clonal type	Type 1		Type 2		Type 3		All types	
Assay	Tg-qPCR1	Tg-qPCR2	Tg-qPCR1	Tg-qPCR2	Tg-qPCR1	Tg-qPCR2	Tg-qPCR1	Tg-qPCR2
P 0.1 GE	17%	25%	8%	25%	33%	67%	19%	39%
LOD95% (95%CI)	1.669 GE (0.87–10.71)	1.943 GE (1.03–10.53)	1.178 GE (0.80–2.68)	2.002 GE (1.06–10.70)	0.351 GE (0.23–1.29)	0.273 GE (0.17–8.40)	1.067 GE (0.77–1.79)	1.561 GE (1.00–3.51)
E% (mean ± SD)	94.4 ± 1.2	95.6 ± 1.8	94.1 ± 1.5	95.1 ± 2.9	93.8 ± 1.0	94.3 ± 0.5	94.1 ± 1.2	95.0 ± 1.9
R^2^(mean)	0,9999	0,9998	0,9999	0,9997	0,9993	0,9999	0,9997	0,9998
LOQ	100 GE	100 GE	100 GE	100 GE	10 GE	10 GE	100 GE	100 GE
Range with Rsd_R_ ≤25%	10^5^–10^2^ GE	10^5^–10^2^ GE	10^5^–10 GE	10^5^–10^2^ GE	10^5^–10 GE	10^5^-1GE	10^5^–10^2^ GE	10^5^–10^2^ GE
PCR performance score	83%	79%	78%	78%	93%	97%	85%	85%

GE, genome equivalents per PCR reaction; P 0.1 GE, detection probability of 0.1 GE/PCR; LOD95%, 95% detection limit as the lowest amount of template DNA which was detected in 95% of the replicates; 95%CI, 95% confidence interval; E%, mean of PCR efficiencies of 4 runs using triplicates; SD, standard deviation; R^2^(mean), averaged R^2^ value over 4 runs, standard curves were generated over the linear dynamic range from 10 to 10^5^ GE/reaction; LOQ, limit of quantification; Rsd_r_, relative repeatability standard deviation; Rsd_R_, relative reproducibility standard deviation; PCR performance score, number of positive replicates/number of total replicates considering all dilution points.

#### 3.1.3 PCR efficiency

Amplification efficiency (E) and the coefficient of determination (R^2^) for both assays were determined for each run and each strain and derived from standard curves covering the linear dynamic range from 10^5^ to 10 GE/reaction.

**Fig. 1:**
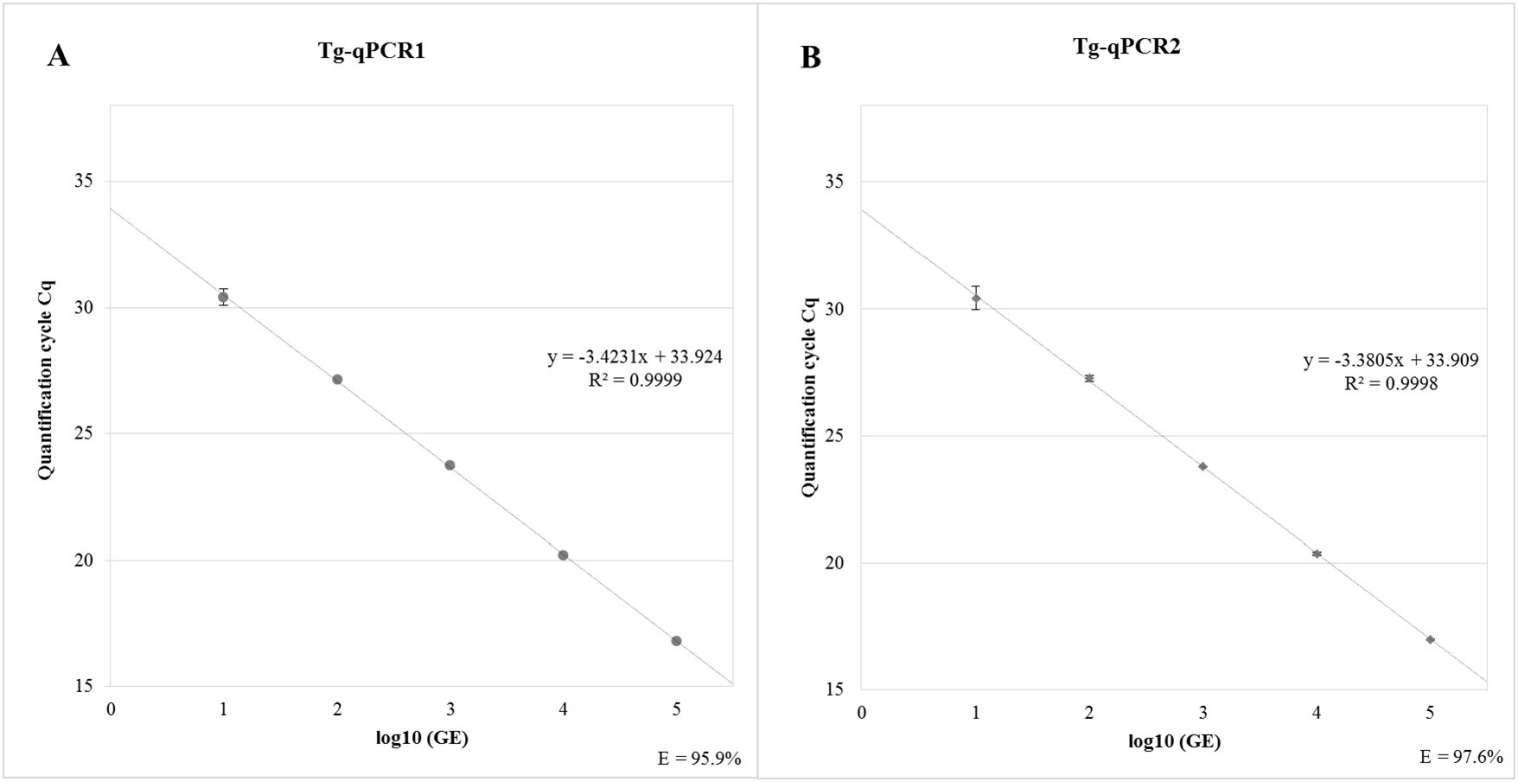
Standard curves of exemplary log-dilution series of genomic DNA of *Toxoplasma gondii* strain ME-49 diluted in 20 ng/μl porcine DNA (10^5^ GE–10 GE/PCR reaction) obtained in Tg-qPCR1 (A) and Tg-qPCR2 (B). The linear equation of each regression line, the coefficient of determination (R^2^), and amplification efficiency (E) are displayed in each graph.

Exemplary standard curves of one run are shown for *T. gondii* ME-49 (Type 2) in Fig. 1. Amplification of the three clonal type strains in both qPCRs showed comparable PCR efficiencies with average PCR efficiencies ranging between 93.8%–94.4% (Tg-qPCR1) and 94.3–95.6% (Tg-qPCR2) (Table 3). Linear regression revealed average R^2^ values for all strains and runs over the 5 log range of 0.9997 (Tg-qPCR1) and 0.9998 (Tg-qPCR2) (Table 3).

#### 3.1.4 Precision & limit of quantification (LOQ)

Considering all clonal type strains, both qPCRs met the required criteria for reliable quantification of Rsd_r_ ≤25% (Broeders et al., 2014; Kralik and Ricchi, 2017) at least over a 4 log range from 10^5^ to 100 GE/reaction (Table 2). The limit of quantification (LOQ) is reached between the log levels 10 and 100 GE/reaction (Table 2, Table 3).

For DNA concentrations ≥100 GE/reaction, both qPCRs show acceptable inter-run reproducibility with Rsd_R_ values of ≤12.7% (Tg-qPCR1) and ≤11.1% (Tg-qPCR2) (Table 2, Table 3).

### 3.2 Performance of the qPCR assays on pork meat samples

Tg-qPCR1 and Tg-qPCR2 showed comparable performance on pork meat samples. All 38 pork meat products were tested negative for *T. gondii* DNA in both qPCRs, while amplification of the IACs could be observed with mean Cq values of 35.8 (±1.3) and 34.5 (±0,8) for CIAC and pUC18, respectively.

Each of the 12 artificially contaminated pork samples tested positive for *T. gondii* DNA with comparable Cq values ≤35 and back-calculated GE numbers (Table 4) in both qPCR assays. Generally, the back-calculated GE numbers per PCR in the spiked samples were four to eleven times less than expected.

**Table 4:** Performance of Tg-qPCR1 and Tg-qPCR2 on spiked pork meat samples.

Sample ID	Spiked GE/25 mg meat^a^	Expected GE/PCR	Tg-qPCR1		Tg-qPCR2	
			Cq_mean_ ± SD^b^	Calculated GE/PCR^c^	Cq_mean_ ± SD^b^	Calculated GE/PCR^c^
16-TO-0002	10^4^	10^3^	27.0 ± 0.0	97	27.7 ± 0.1	87
16-TO-0003	10^4^	10^3^	27.1 ± 0.1	94	27.6 ± 0.2	83
16-TO-0007	10^4^	10^3^	26.7 ± 0.2	118	27.1 ± 0.1	117
16-TO-0008	10^4^	10^3^	25.5 ± 0.0	269	25.8 ± 0.0	268
16-TO-0004	10^3^	10^2^	28.9 ± 0.1	n.a.	29.1 ± 0.1	n.a.
16-TO-0005	10^3^	10^2^	30.5 ± 0.0	n.a.	30.7 ± 0.4	n.a.
16-TO-0006	10^3^	10^2^	29.6 ± 0.2	n.a.	30,1 ± 0.1	n.a.
16-TO-0007	10^3^	10^2^	30.2 ± 0.2	n.a.	30.8 ± 0.1	n.a.
16-TO-0002	10^2^	10^1^	32.8 ± 0.0	n.a.	33.3 ± 0.3	n.a.
16-TO-0003	10^2^	10^1^	35.3 ± 1.7	n.a.	34.1 ± 1.2	n.a.
16-TO-0005	10^2^	10^1^	33.9 ± 0.2	n.a.	33.8 ± 2.2	n.a.
16-TO-0006	10^2^	10^1^	34.1 ± 0.4	n.a.	34.1 ± 1.1	n.a.

GE/PCR, number of genome equivalents per PCR reaction; Cq, quantification cycle; SD, standard deviation; n.a., not applicable.

a Number of genome equivalents of *T. gondii* strain ME-49 spiked in 25 mg meat samples.

b Mean and SD of duplicate measurements.

c GE per PCR were quantified using standard curves from 10^5^ to 10 GE/PCR. Calculation for samples spiked with ≤10^3^GE/25 mg meat was not valid, as Cq values were below the limit of quantification.

### 3.3 Analytical sensitivity and specificity of the 529 RE conventional endpoint PCR

To determine the analytical sensitivity of the cPCR, dilution series of 1 ng to 1 fg of genomic DNA of *T. gondii* RH in water were subjected to analysis. In 4/4 experiments, DNA of *T. gondii* strain RH could be detected with a high sensitivity at concentrations as low as 100 fg DNA, which corresponds to 1.41 GE per PCR reaction.

However, this cPCR using TOX5/Tox-8 as a primer pair was not suitable for the detection of *T. gondii* in pork meat samples in our hands as this PCR generated unspecific amplicons. When dilutions of genomic *T. gondii* DNA in porcine DNA, as well as pork meat samples were subjected to PCR analysis, non-specific bands with similar size (472 bp) to the desired *T. gondii*-specific PCR product (452 bp) were observed. Sanger-sequencing of these non-specific products followed by BLAST analysis revealed cross-amplification of porcine DNA present in different *Sus scrofa* breeds with this cPCR (data not shown). Attempts to eliminate this non-specific amplification by gradually increasing the annealing temperature from 60 °C of up to 67 °C and/or by reducing the duration of the final extension step from 10 min to 2 min showed no satisfactory results (data not shown). Further optimization experiments have not been conducted.

## 4 Discussion

In recent literature, the 529 RE has evolved as the common target of choice for molecular detection of *T. gondii* DNA, as its high copy number of 200–300 enables high sensitivity (Schares et al., 2008; Talabani et al., 2009; Herrmann et al., 2010; Opsteegh et al., 2010; Sterkers et al., 2010; Gomez-Samblas et al., 2015; Opsteegh et al., 2016; Krücken et al., 2017; Schares et al., 2017; Burrells et al., 2018; Friedrich-Loeffler-Institut, 2018; Schares et al., 2018). This study aimed to validate and compare three molecular methods targeting the 529 RE to detect *T. gondii* DNA in pork meat samples. Tg-qPCR1 was originally described by Reischl et al. (2003) for highly sensitive and specific detection of *T. gondii* DNA using a pair of FRET hybridization probes on a LightCycler instrument (Reischl et al., 2003). The qPCR was adapted by Opsteegh et al. (2010) using a modified TaqMan™ probe and an altered cycling protocol in addition to the incorporation of an competitive internal amplification control (CIAC) (Opsteegh et al., 2010). In both publications, a high sensitivity with detection limits between 16 and 20 fg DNA was described, which corresponds to 0.23-0-28 GE according to our calculation. In this study, the protocol was further adapted for application on an ABI 7500 system, which could explain the decreased sensitivity observed in our experiments. Although, we were also able to detect *T. gondii* DNA at low concentrations of up to 0.1 GE/PCR in 19% of the replicates, the 95% detection limit for all three clonal type strains was reached at higher concentrations of 1.067 GE/PCR.

Tg-qPCR2 was originally described by Talabani et al. (2009) to diagnose human ocular toxoplasmosis and has been used to amplify *T. gondii* DNA in ocular or amniotic fluid (Sterkers et al., 2010; Robert-Gangneux et al., 2017). More recently, Tg-qPCR2 has additionally been used to detect *T. gondii* tissue cysts in chicken (Opsteegh et al., 2016; Schares et al., 2017; Schares et al., 2018) but has not been validated in detail before. In the original publication, a non-competitive internal amplification control (NCIAC) was included, but not further specified. As the addition of an IAC has been proposed to be mandatory for molecular detection of foodborne pathogens (Hoorfar et al., 2003), we added plasmid pUC18 as target for a NCIAC to Tg-qPCR2, which has already been successfully used in PCRs for detection of *Bacillus cereus* (Frentzel et al., 2018), *Yersinia enterocolitica* (Mäde et al., 2008) and *Salmonella* spp. (Anonymous, 2013).

The direct comparison of Tg-qPCR1 and Tg-qPCR2 in this study revealed that both qPCRs show comparable performance on dilution series of the three clonal type strains of *T. gondii* in porcine DNA as well as for detection of *T. gondii* DNA in pork meat samples. The analytical sensitivity of Tg-qPCR2 was slightly higher based on a higher detection probability at low concentrations of 0.1 GE/PCR. A reason for this could be the use of a locked nucleic acid (LNA) probe, which can enhance the fluorescence signal and increase specificity and sensitivity of qPCR assays in comparison to standard TaqMan™ probes (Reynisson et al., 2006). Among others, a further reason for the lower analytical sensitivity of Tg-qPCR1 could be a slight inhibition at low concentrations of target-DNA due to the use of a competitive IAC. Both qPCR assays proved to be highly specific for detection of *T. gondii* as none of the other tested non-target parasites were detected and no cross-reactivity to DNA extracted from pork meat samples was observed. Additionally, both qPCR assays allow reliable quantification of *T. gondii* DNA in pork over at least 4 log ranges from 10^5^ to 100 GE/PCR and thus determination of the parasitic load in diverse tissues compared to the cPCR or bioassay.

In comparison to the bioassay as gold standard for *T. gondii* detection in meat samples, where several hundred gram of meat are analyzed, molecular methods are generally considered to be less sensitive, as a significantly lower amount of tissue is used for DNA extraction (about 25 mg). However, comparable sensitivity can be achieved when molecular detection is combined with a pepsin or trypsin digestion or magnetic capture to analyze larger sample sizes (Schares et al., 2018).

Taking together, this study showed that both qPCRs, Tg-qPCR1 and Tg-qPCR2 targeting the 529 RE are similarly suited for the detection of *T. gondii* DNA in pork meat samples. In contrast to the two qPCR assays, the cPCR with primer pair TOX5/Tox-8 was not suitable for detection of *T. gondii* DNA in pork, as unspecific amplification of porcine DNA was observed. Nevertheless, the described cPCR showed a high sensitivity with a detection limit of 100 fg corresponding to 1.41 GE/PCR. Thus, it could potentially be used for the examination of other food matrices or animal tissues and has e.g. already been applied to detect *T. gondii* oocysts in fecal samples of cats (Schares et al., 2008; Herrmann et al., 2010). The results underline the importance of validating molecular methods in the presence of specified matrix DNA, before screening of large sample numbers is performed.

## References

[bb0005] Anonymous (2013). Microbiology of Food and Animal Feeding Stuffs - Polymerase Chain Reaction (PCR) for the Detection of Food-borne Pathogens - Method for the Detection of Salmonella (DIN 10135:2013-05).

[bb0010] Broeders S., Huber I., Grohmann L., Berben G., Taverniers I., Mazzara M. (2014). Guidelines for validation of qualitative real-time PCR methods. Trends Food Sci. Technol..

[bb0015] Burrells A., Taroda A., Opsteegh M., Schares G., Benavides J., Dam-Deisz C. (2018). Detection and dissemination of *Toxoplasma gondii* in experimentally infected calves, a single test does not tell the whole story. Parasit. Vectors.

[bb0020] Bustin S.A., Benes V., Garson J.A., Hellemans J., Huggett J., Kubista M. (2009). The MIQE guidelines: minimum information for publication of quantitative real-time PCR experiments. Clin. Chem..

[bb0025] BVL (2016). Leitlinien zur Einzellabor-Validierung qualitativer real-time PCR Methoden.

[bb0030] Cook A.J., Gilbert R.E., Buffolano W., Zufferey J., Petersen E., Jenum P.A. (2000). Sources of *Toxoplasma* infection in pregnant women: European multicentre case-control study. European Research Network on Congenital Toxoplasmosis. BMJ.

[bb0035] Costa J.-M., Bretagne S. (2012). Variation of *B1* gene and AF146527 repeat element copy numbers according to *Toxoplasma gondii* strains assessed using real-time quantitative PCR. J. Clin. Microbiol..

[bb0040] Dzib Paredes G.F., Ortega-Pacheco A., Rosado-Aguilar J.A., Acosta-Viana K.Y., Guzmán-Marín E., Jiménez-Coello M., Makun H. (2016). *Toxoplasma gondii* in meat for human consumption – a brief review of the most described strategies for its detection and quantification. Significance, Prevention and Control of Food Related Diseases.

[bb0045] Frentzel H., Thanh M.D., Krause G., Appel B., Mader A. (2018). Quantification and differentiation of Bacillus cereus group species in spices and herbs by real-time PCR. Food Control.

[bb0050] Friedrich-Loeffler-Institut (2018). Toxoplasma gondii. Amtliche Methodensammlung: Meldepflichtige Tierkrankheiten.

[bb0055] Gomez-Samblas M., Vilchez S., Racero J.C., Fuentes M.V., Osuna A. (2015). Quantification and viability assays of *Toxoplasma gondii* in commercial “Serrano” ham samples using magnetic capture real-time qPCR and bioassay techniques. Food Microbiol..

[bb0060] Herrmann D.C. (2012). Molecular Typing of *Toxoplasma gondii* Isolates From Cats and Humans in Germany.

[bb0065] Herrmann D.C., Pantchev N., Vrhovec M.G., Barutzki D., Wilking H., Frohlich A. (2010). Atypical *Toxoplasma gondii* genotypes identified in oocysts shed by cats in Germany. Int. J. Parasitol..

[bb0070] Homan W.L., Vercammen M., De Braekeleer J., Verschueren H. (2000). Identification of a 200- to 300-fold repetitive 529 bp DNA fragment in *Toxoplasma gondii*, and its use for diagnostic and quantitative PCR. Int. J. Parasitol..

[bb0075] Hoorfar J., Cook N., Malorny B., Wagner M., De Medici D., Abdulmawjood A. (2003). Making internal amplification control mandatory for diagnostic PCR. J. Clin. Microbiol..

[bb0080] Kapperud G., Jenum P.A., Stray-Pedersen B., Melby K.K., Eskild A., Eng J. (1996). Risk factors for *Toxoplasma gondii* infection in pregnancy. Results of a prospective case-control study in Norway. Am. J. Epidemiol..

[bb0085] Kralik P., Ricchi M. (2017). A basic guide to real time PCR in microbial diagnostics: definitions, parameters, and everything. Front. Microbiol..

[bb0090] Krücken J., Blümke J., Maaz D., Demeler J., Ramünke S., Antolová D. (2017). Small rodents as paratenic or intermediate hosts of carnivore parasites in Berlin, Germany. PLoS One.

[bb0095] Liu Q., Wang Z.D., Huang S.Y., Zhu X.Q. (2015). Diagnosis of toxoplasmosis and typing of *Toxoplasma gondii*. Parasit. Vectors.

[bb0100] Mäde D., Reiting R., Strauch E., Ketteritzsch K., Wicke A. (2008). A real-time PCR for detection of pathogenic *Yersinia enterocolitica* in food combined with an universal internal amplification control system. J. Verbr. Lebensm..

[bb0105] Opsteegh M., Langelaar M., Sprong H., Den Hartog L., De Craeye S., Bokken G. (2010). Direct detection and genotyping of *Toxoplasma gondii* in meat samples using magnetic capture and PCR. Int. J. Food Microbiol..

[bb0110] Opsteegh M., Schares G., Blaga R., Van Der Giessen J. (2016). Experimental Studies on *Toxoplasma gondii* in the Main Livestock Species (GP/EFSA/BIOHAZ/2013/01) Final Report.

[bb0115] Reischl U., Bretagne S., Kruger D., Ernault P., Costa J.M. (2003). Comparison of two DNA targets for the diagnosis of Toxoplasmosis by real-time PCR using fluorescence resonance energy transfer hybridization probes. BMC Infect. Dis..

[bb0120] Reynisson E., Josefsen M.H., Krause M., Hoorfar J. (2006). Evaluation of probe chemistries and platforms to improve the detection limit of real-time PCR. J. Microbiol. Methods.

[bb0125] Robert-Gangneux F., Darde M.L. (2012). Epidemiology of and diagnostic strategies for toxoplasmosis. Clin. Microbiol. Rev..

[bb0130] Robert-Gangneux F., Brenier-Pinchart M.-P., Yera H., Belaz S., Varlet-Marie E., Bastien P. (2017). Evaluation of toxoplasma ELITe MGB real-time PCR assay for diagnosis of toxoplasmosis. J. Clin. Microbiol..

[bb0135] Scallan E., Hoekstra R.M., Mahon B.E., Jones T.F., Griffin P.M. (2015). An assessment of the human health impact of seven leading foodborne pathogens in the United States using disability adjusted life years. Epidemiol. Infect..

[bb0140] Schares G., Vrhovec M.G., Pantchev N., Herrmann D.C., Conraths F.J. (2008). Occurrence of *Toxoplasma gondii* and *Hammondia hammondi* oocysts in the faeces of cats from Germany and other European countries. Vet. Parasitol..

[bb0145] Schares G., Herrmann D.C., Maksimov P., Matzkeit B., Conraths F.J., Moré G. (2017). Chicken line-dependent mortality after experimental infection with three type IIxIII recombinant *Toxoplasma gondii* clones. Exp. Parasitol..

[bb0150] Schares G., Koethe M., Bangoura B., Geuthner A.C., Randau F., Ludewig M. (2018). *Toxoplasma gondii* infections in chickens - performance of various antibody detection techniques in serum and meat juice relative to bioassay and DNA detection methods. Int. J. Parasitol..

[bb0155] Sterkers Y., Varlet-Marie E., Cassaing S., Brenier-Pinchart M.P., Brun S., Dalle F. (2010). Multicentric comparative analytical performance study for molecular detection of low amounts of *Toxoplasma gondii* from simulated specimens. J. Clin. Microbiol..

[bb0160] Talabani H., Asseraf M., Yera H., Delair E., Ancelle T., Thulliez P. (2009). Contributions of immunoblotting, real-time PCR, and the Goldmann-Witmer coefficient to diagnosis of atypical toxoplasmic retinochoroiditis. J. Clin. Microbiol..

[bb0165] Tenter A.M., Heckeroth A.R., Weiss L.M. (2000). *Toxoplasma gondii*: from animals to humans. Int. J. Parasitol..

[bb0170] WHO (2015). WHO Estimates of the Global Burden of Foodborne Diseases: Foodborne Disease Burden Epidemiology Reference Group 2007–2015.

